# P054. Chronic migraine and onabotulinumtoxinA: results from clinical practice

**DOI:** 10.1186/1129-2377-16-S1-A117

**Published:** 2015-09-28

**Authors:** Matteo Paolucci, Riccardo Altavilla, Giulia Gambale, Claudio Altamura, Giovanna Viticchi, Marco Bartolini, Mauro Silvestrini, Fabrizio Vernieri

**Affiliations:** Headache Centre, Policlinico Universitario Campus Bio-Medico, Rome, Italy; Clinica Neurologica, Università Politecnica delle Marche, Ancona, Italy

## Background

OnabotulinumtoxinA injection according to PREEMPT protocol is a second-line therapy for chronic migraine (CM)[1]. While its efficacy on frequency of headache has been demonstrated[2], our current clinical experience indicates that patients report benefits regardless of reduction of frequency of attacks, as Lipton et al previously showed[3]. The present study aimed at assessing the impact of botulin injection on intensity, quality and perception of pain in patients with CM.

## Materials and methods

We enrolled 25 patients who underwent botulin injections from April 2014 to June 2015. We evaluated patients at baseline (T0, the day of the first botulin injection) and every three months, along with a new injection session. Evaluation consisted in a multiparametric self-assessment questionnaire: pain intensity through 11 point Box Scale (BS-11) and Present Pain Intensity (PPI), changes in functioning through 6-point Behavioral Rating Scale (BRS-6), quality of pain through Short-form McGill Pain Assessment Questionnaire (SF-MPQ) and disability through Migraine Disability Assessment (MIDAS) questionnaire and HIT-6.

At the present time, we have follow-up data for 57% of patients at three months (T1) and for 38% of patients at six months (T2).

## Results

We found a global reduction in the intensity of perceived pain and in restriction of activities due to headache: Wilcoxon signed-rank test showed significant reduction in median values of BS-11 at T1 (p 0.026) and T3 (p 0.017), of PPI at T1 (p 0.02) and T3 (p 0.046), of BRS-6 at T1 (p 0.008) and T3 (0.026). Analysis of SF-MPQ showed a significant reduction in median values of the descriptors “throbbing” at T1 (p 0.041), “splitting” at T1 (p 0.041) and “tiring-exhausting” at T1 (p 0.011). We did not find significant difference in MIDAS score at T1 and T3 and in frequency of headache in the past three months.

## Conclusions

Our preliminary results show that the OnabotulinumtoxinA usefulness is primarily in improving the quality of life more than in reducing the frequency of headache.

Written informed consent to publication was obtained from the patient(s).
